# Impact of equivalent units of production on state-controlled unit cost calculation for fair pricing of pharmaceuticals: a scoping review

**DOI:** 10.1080/20523211.2025.2564820

**Published:** 2025-10-23

**Authors:** Salem Udoh, Tomasz Wnuk-Pel

**Affiliations:** a Department of Finance, Tomas Bata University, Zlin, Czech Republic; b Department of Accounting, Faculty of Management, University of Lodz, Łódź, Poland; c Department of Accounting and Logistics, Linnaeus University, Växjö, Sweden

**Keywords:** Equivalent units of production, unit costs of pharmaceuticals, fair pricing of pharmaceuticals, scoping review

## Abstract

**Background:**

Ninety-one percent of 1500 patient groups surveyed across 78 countries perceive pharmaceutical firms’ pricing policies as unfair. Despite this, there is little evidence of pharmaceutical companies adopting continuous process costing, a management accounting method that could help transparently track costs and determine fair pricing. This study investigates the link between unit costs, equivalent production units, and fair pharmaceutical pricing. To enhance transparency, fairness, and affordability, we propose mandating the disclosure of unit cost formulas, costing methods, and markup ceilings in annual reports, in alignment with UN SDG goals.

**Methods:**

This review followed the Joanna Briggs Institute’s (JBI) methodology for scoping reviews to frame the research question, identify relevant studies in databases, select studies, extract the data, report the results and guide consultation sessions with stakeholders with lived experience on potential implications. The search period was January 2022 to November 2023. We used Preferred Reporting Items for Systematic Reviews and Meta Analysis (PRISMA)-Scoping Review extension to present the results.

**Results:**

46 articles were eligible out of 1,281 initially screened. 8 articles addressed pharmaceutical unit production costs; 1 empirical study focused on Equivalent Units of Production (EUP) practices, revealing discrepancies between industry costing practices and academic models; and, the remaining 37 articles explored fair pricing frameworks, emphasising value-based pricing, ethics, and policy considerations. Three gaps emerged: no studies link pharmaceutical pricing to continuous process costing/EUP, despite extensive fair pricing research; absence of standardised methodologies for applying continuous costing in pharmaceutical contexts; and lack of state-regulated uniform costing systems or enforceable mark-up ceilings, impeding cost transparency and fair pricing.

**Conclusions:**

No evidence linked pharmaceutical pricing models to continuous process costing/EUP. Addressing this gap requires mandatory disclosures of regulated uniform costing methods and mark-up ceilings in published annual financial reports. This will improve transparency, social accountability, fair pricing, and medicine affordability – aligning with UN SDGs 3 (Good Health) and 10 (Reduced Inequalities).

## Introduction

### Background

The rising cost of medicines has become a major concern for countries aiming to provide universal healthcare, as highlighted in the UN Sustainable Development Goals (SDGs). This makes it important to better understand how drug prices are set – especially how production costs, pricing methods, and efficiency in manufacturing all come together to influence final prices (Andersson & McMenamin, 1992; Bendicksen et al., 2021; Drury & Tayles, 1994; Wilensky, 2016). Drug prices depend on many factors, such as how essential a medicine is, whether it's under patent, how much competition exists, the rules set by national regulators (Cohn, 2016; Syversen et al., 2024) as well as costing and pricing methods (Modell, 2014; Skinner, 1970; Van der Stede, 2011). While many studies have explored large-scale pricing trends (Babar, 2022), how governments buy medicines and critiques of profit margins (Basu et al., 2008; Towse et al., 2018), others have looked at how manufacturing and technology affect costs (Berlak & Götz, 2021; Pinheiro et al., 2006; Zawawi & Hoque, 2008).

### Gaps in literature

However, less attention has been paid to the basic methods used to calculate the actual cost of making each unit of medicine. In particular, the concept of *equivalent units of production* (EUP), used in continuous process costing, has been overlooked – despite being essential in industries like pharmaceuticals where the production process is continuous and involves work-in-progress inventory (Amaral & Guerreiro, 2019; Drury, 2021). EUP helps ensure that costs are fairly and accurately assigned to each unit produced.

While value-based and cost-plus pricing models dominate industry discourse (Babar, 2015, 2022; Skinner, 1970), the pharmaceutical sector’s opacity around costing practices – compounded by proprietary claims and information asymmetry – has hindered oversight and public trust (World Health Organization [WHO], 2020). The absence of standardised costing conventions has also led to inconsistent profitability outcomes and skepticism over pricing fairness (Babar, 2022; Vogler, 2018; Vogler et al., 2015).

### Aim/objective

This study addresses these gaps by proposing a new approach: integrating EUP-driven continuous process costing within government-regulated frameworks to minimize arbitrary mark-ups and prioritize societal welfare (social accountability) over profit maximization motives. Through a scoping review methodology (Arksey & O'Malley, 2005), we synthesize evidence on EUP's role in pharmaceutical costing and pricing, drawing on Actor-Network Theory (ANT)^1^ (Latour, 1987) to position management accounting as a critical actor in shaping fair pricing outcomes. Rather than seeing pricing as purely profit-driven, ANT presents it as the result of interactions among economic, legal, technical, and human elements. The study aims to (1) promote a more transparent and fair pricing model using EUP principles, promote a more transparent and fair pricing model using EUP principles, and (2) support regulatory changes that link production costs with public interest-helping balance business sustainability with universal access to healthcare, in line with growing calls for fairer drug pricing (Babar, 2022; Van der Stede, 2011).^2^

## Methods

This review was conducted following the Joanna Briggs Institute’s (JBI) methodology for scoping reviews (Peters et al., 2015) and included input from management accounting academics and experts – people with lived experience. The involvement of these experts/stakeholders in scoping reviews aims to provide grounding for the study and to foster discussion regarding potential implications (Arksey & O’Malley, 2005; Daudt et al., 2013; Levac et al., 2010).

### Review question

What evidence exists regarding whether pharmaceutical unit production costs incorporate equivalent units of production (EUP) under continuous process costing as a basis for unit cost calculation and setting fair pricing?

### Inclusion criteria

Studies on unit costs of production, equivalent units of production and fair pricing of pharmaceuticals respectively are included (Tables 1 and 2) on the premise that a fair unit selling price for pharmaceuticals stems from a lower unit production cost under the continuous process costing method but within the framework of a regulated uniform costing system. This is because unit cost underpins all pricing methods, according to Drury (2021), with ‘fair’ defined contextually. In this study’s context, fair pricing is essentially viewed as an extension of the ‘true and fair concept’ of auditing, emphasising verifiability. Consequently, articles that do not align with the review question were excluded (Table 3).

**Table 1.** Included studies – Summary of sources that discussed unit cost of production concept and equivalent units of production (unit cost of production, *n* = 8; equivalent units of production, *n* = 1).

Author(s)	Summaries
**Costs of production (n** **=** **8)**	
**[1]** Hill et al. (2018)	**Title:** Estimated costs of production and potential prices for the WHO essential medicines list. **Method:** Price estimation formula (formulation, packaging, tax, 10% profit) based on Indian API prices. **Findings**: Prices ranged from $0.01–$1.45/unit; accurate for HIV/TB/malaria drugs.
**[2]** Towse et al. (2018)	**Title**: Critique of production cost estimates for WHO Essential Medicines List. **Method:** Critique of existing models. **Findings:** Argues for competitive global procurement to reduce generic drug prices.
**[3]** Basu et al. (2008)	**Title**: Analysis of manufacturing costs in pharmaceutical companies. **Method:** Annual report data analysis. **Findings:** Brand-name companies show inverse correlation between COGS and R&D spending as suggested by Vernon’s theory.
**[4]** Pinheiro et al. (2006)	**Title**: Production costs of antiretroviral drugs. **Method:** Brazilian Actual Pharmaceutical Ingredients (API) cost data + WHO survey. **Findings:** Demonstrates feasibility of price reductions for antiretroviral (ARVs) drugs in developing markets.
**[5]** Drury and Tayles (1994)	**Title**: Product costing in UK manufacturing organisations. **Method:** Theoretical framework. **Findings:** Prices are based on unit cost calculations.
**[6]** Edwards et al. (2003)	**Title**: Costing, pricing and politics in the British steel industry, 1918–1967. **Method:** Review of literature. **Findings:** Highlights challenges of uniform costing systems (e.g. data asymmetry). Key takeaways for pharma industry, despite study’s age.
[7] Berlak and Götz (2021)	**Title**: Concept for the urban production of pharmaceuticals to compensate for local shortages. **Method:** Conceptual proposal **Findings:** Suggests small-scale urban facilities for tablet manufacturing.
**[8]** Bischof (1996)	**Title**: Pharmaceuticals in the era of cost-containment. **Method:** Policy analysis. **Findings:** Rising drug costs linked to new production technologies.
**Equivalent units of production (n** **=** **1)**	
**[1]** Guerreiro and Amaral (2018)	**Title**: Equivalent units of production: a new look at an old issue. **Method:** Qualitative exploratory survey on 175 top Brazilian companies. **Findings:** No objective method for completion rate calculation, but practice aligns with theory.

Source: Authors.

**Table 2.** Included studies – summary of sources that discussed fair pricing (n = 37).

Key studies on fair pricing	
Author(s)	Summaries
**[1]** Guerreiro and Amaral (2018)	**Title:** Cost-based price and value-based price: are they conflicting approaches? **Method:** Qualitative case study (Brazil). **Findings:** The cost-plus margin formula does not necessarily imply cost-based price setting.
**[2]** Amaral and Guerreiro (2019)	**Title:** Factors explaining a cost-based pricing essence. **Method:** Survey of Brazilian pricing professionals. **Findings:** Margins reflect competition/value, not just cost.
**[3]** Davenport et al. (2012)	**Title:** Capturing the value of pricing analytics. **Method:** Book chapter review. **Findings:** Analytics reveal value and optimise pricing decisions.
**[4]** Vogler et al. (2016)	**Title:** Policy interventions related to medicines: Survey of measures taken in European countries during 2010–2015. **Method:** Survey of 32 European countries. **Findings:** Price reductions/freezes were most common (557 measures).
**[5]** World Health Organization (2020)	**Title:** WHO guideline on country pharmaceutical pricing policies. **Method:** Policy guidelines. **Findings:** High drug prices challenge both high- and low-income nations.
**[6]** Scherer (2000)	**Title:** The pharmaceutical industry. **Method:** Handbook analysis. **Findings:** Generic market share depends on regulations, insurer strategies.
**[7]** Suleman and Gray (2017)	**Title:** New business models for research and development with affordability requirements are needed to achieve fair pricing of medicines. **Method:** Editorial. **Findings:** Push (direct funding), pull (incentives rewards to breakthrough) and pool (information sharing) implications considered.
**[8]** Moon et al. (2020)	**Title:** Defining the concept of fair pricing for medicines. **Method:** Editorial analysis. **Findings:** Fairness balances demand (access) and supply (innovation).
**[9]** Walley (2007)	**Title:** Fair pricing for medicines in the UK. **Method:** Editorial. **Findings:** UK prices higher than EU; PPRS fails to link prices to patient value.
**[10]** Skinner (1970)	**Title:** The determination of selling prices. **Method:** A questionnaire survey. **Findings:** 80% of firms use cost-plus pricing, covering overheads.
**[11]** Broekhof (2002)	**Title:** Transparency in the pharmaceutical industry – A cost accounting approach to the prices of drugs. **Method:** Industry cost analysis. **Findings:** Pharma lacks advanced cost accounting, leading to high indirect costs.
**[12]** Schreyögg et al. (2006)	**Title:** Cost Accounting to determine prices: How well do prices reflect costs in the German DRG system? **Method:** Hospital data review. **Findings:** Poor documentation of case-related costs; accounting reforms needed.
**[13]** Spinello (1992)	**Title:** Ethics, pricing and the pharmaceutical industry. **Method:** Rawls’ justice framework. **Findings:** Distributive justice should guide drug pricing.
**[14]** Babar (2022)	**Title**: Forming a Medicines Pricing Policy for low and middle-income countries (LMICs): The case for Pakistan. **Method:** Survey of 100,000 respondents. **Findings**: Mark-up regulations exist but need value-based pricing expertise.
**[15]** Babar et al. (2007)	**Title**: Evaluating drug prices, availability, affordability and price components: Implications for access to drugs in Malaysia. **Method:** The World Health Organization and Health Action International methodology. **Findings**: High end-user prices due to unregulated markups.
**[16]** Vogler and Martikainen (2015)	**Title**: Pharmaceutical pricing in Europe. **Method:** Case study-30 countries. **Findings**: Pricing policies focus on ex-factory and supply-chain levels.
**[17–37] Various authors**	**Title:** Drug prices and incentives to innovation by the pharmaceutical industry. **Method:** Case studies, policy reviews. **Findings:** Rising prices driven by monopolies, aging populations, and weak regulation. Expanded summary. Key themes: Challenges: High drug costs persist despite regulations (e.g. mandatory price cuts, generic disclosure). Drivers: Monopoly pricing, aging populations, and regulatory gaps (e.g. non-reimbursable drugs). Solutions: Successful models: New Zealand’s monopsony purchasing. Barriers: Transparency issues, anti-competitive practices, and declining R&D productivity.

Source: Authors.

**Table 3.** Excluded studies criteria.

	Title
**EUP concept (n** **=** **7)**	Teaching Equivalent Production with a Chart
	Reviews on Accounting of Revenues Associated to the Production Cost of the Work in Progress
	The Unit Cost Denominator in Process Costing
	Cost Flow Diagrams as an Alternative Method of External Problem Representation – A Diagrammatic Approach to Teaching Cost Accounting and Evidence of Its Effectiveness
	Cost Accounting Teaching: Focus on the Processes and Operations
	A Note on Equivalent Units Calculations
	Closing Pandora’s Box: Reducing Students’ Confusion with A Process Costing Simulation
**Others (1228)**	Pricing reports (n = 3)
	Regulations (n = 16)
	Pharmacoeconomics studies (n = 2)
	Pricing policies studies (n = 18)
	Primary care studies (n = 9)
	Reimbursement studies (n = 6)
	Impact of competition on prices studies (n = 5)
	Comparative studies (n = 24)
	Audit and feedback studies (n = 15)
	Technical analysis studies (n = 10)
	Quality improvement studies (n = 2)
	Price referencing studies (n = 33)
	Cost-containment studies (n = 4)
	Research and development cost studies (n = 4)
	Other ineligible contexts (n = 1008)
	Duplicates (n = 76)

Source: Authors.

### Participants

This scoping review considered all research studies and policy papers that included equivalent units of production, cost of production in the pharmaceutical industry, fair pricing of pharmaceuticals, value-based pricing of pharmaceuticals and cost-based pricing for the industry. The inclusion criteria were expanded in accordance with this study’s protocol to include studies addressing pricing policies, manufacturing costs, estimated production costs, proposed drug prices and critiques due to inconsistent definitions and applications in the literature.

### Concepts

In this study, there are three concepts of interest: equivalent units of production, unit cost of equivalent production and fair pricing of pharmaceuticals which need clarification. These cost terms are associated with continuous process costing for the manufacturing of homogenous products such as pharmaceuticals.

**Figure 1.**
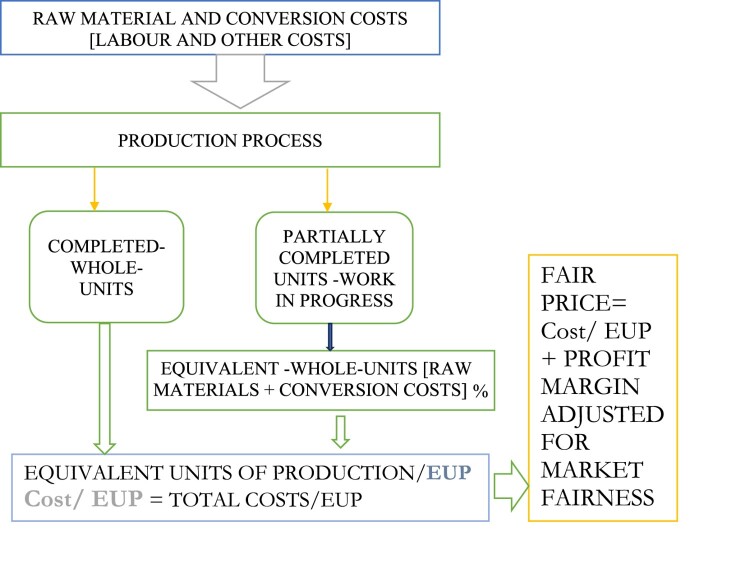
Visual flow explanation of the equivalent units of production process: raw materials and labour 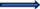
production. Source: Authors.

**Equivalent Units of Production (EUP):** In a manufacturing process of homogenous products, EUP represents the sum of completed units plus the respective fully completed units’ equivalence for materials and conversion costs – labour and overheads- in work-in-process (partially completed units). Unit cost of equivalent production is the sum of respective cost per equivalent unit for material and conversion costs. These concepts help in valuing both finished goods and work in process inventory (cf., Figure 1).

**Fair Pricing:** A verifiable, cost-plus-derived price aligned with auditing’s ‘true and fair' principle. Moreover, for clarity we present below a conceptual framework linking the key concepts together.

The process begins with introduction of the raw materials and conversion costs – labour costs and overheads – into the production process. The process subsequently produces completed units and partially completed units (work-in-progress). For the purpose of calculation of accurate unit cost, equivalent completed units in the work-in-progress are calculated for raw materials and conversion costs and added to the completed to derive EUP. The cost per EUP is marked up after adjusting for market fairness to derive a fair price.

### Context

This review considered studies that focused on the manufacturing and pricing of pharmaceuticals. Studies conducted in all countries without time limits were eligible for inclusion.

### Types of sources

For this scoping review, published and unpublished original research and policy papers were included that explored issues related to equivalent units of production, unit cost of equivalent production, and fair pricing of pharmaceuticals.

### Search strategy

The search strategy aimed to find both published and unpublished literature including policy papers, theses and dissertations. A six-step search strategy was used to identify published literature. An initial limited search of Google Scholar, Zenodo and Social Science Research Network (SSRN, Elsevier) was undertaken on the identified keywords ‘equivalent units of production of/for pharmaceuticals/drugs, medicines’ and ‘fair pricing of pharmaceuticals’. This search was followed by an analysis of the text words contained in the title and abstract and of the index terms used to describe the article. A second systematic search using all identified keywords and index terms was then undertaken across all included published literature databases up to December 17, 2022. Meanwhile, the databases searched for published literature included Web of Science, Scopus, and ScienceDirect (Elsevier), and APA PsycINFO (EBSCO). Third was the search for grey literature completed on December 17, 2022, and targeted the following websites and digital repositories: conference proceedings, digital dissertations, Google Scholar, Gray LIT Network, Gray Literature Report (via New York Academy of Medicine website), Gray Source: A Selection of Web-based Resources in Gray Literature, Index to Theses, ProQuest Dissertations and Theses Databases, and TRIP (Turning Research into Practice). Fourth, reference lists of included literature were hand-searched for additional relevant studies. Fifth, in May 2023, the ‘Publish and Perish’ search engine tool was utilised to search Crossref on unit cost of production for pharmaceuticals which yielded 929 articles from the entire web. Additionally, the searches were crosschecked on PubMed, Scopus, and Google Scholar, but no articles were found. Subsequently, the 929 articles were screened based on the inclusion criteria to only two extra relevant titles. A thorough review of the full texts of these studies was conducted to assess their relevance and excluded articles that primarily focused on pedagogical aspects related to equivalent units. The studies that met the inclusion criteria are presented in Tables 1 and 2. Concerning fair pricing, the findings presented in Table 2 were observed. Finally, in November 2023 twenty-one extra sources were recommended by reviewers for inclusion in accordance with Table 2. The search strategy is included in Appendices 1–2 in the Supplemental Material.

### Study selection

After completing the search, citations were uploaded to Zotero for de-duplication and then imported into JBI SUMARI. Two reviewers independently screened titles/abstracts and selected studies meeting inclusion criteria. Full-text articles were uploaded to SUMARI and reassessed by both reviewers for eligibility. Disagreements at any stage were resolved via consensus or Lead author’s veto. Consistent with scoping review methodology (Arksey & O’Malley, 2005), no quality appraisal was performed.

To minimise discrepancies, a structured a five-step approach was implemented: consensus on inclusion/exclusion criteria, lead-author screening, second-reviewer verification, joint resolution of mismatches, and inter-rater reliability (IRR) calculation (Cohen’s κ = 0.7). Disagreements occurred in 30% of studies, reflecting ‘substantial' to ‘almost perfect' agreement (Landis & Koch, 1977).

### Data extraction

Following the JBI scoping review methodology (Peters, 2015), data were extracted from included papers by two independent reviewers using a data extraction tool (Appendix 2 in the Supplemental Material) developed by the reviewers and refined following piloting with a small number of studies and subsequently applied to all included studies. Categories of studies were refined throughout the data extraction process to ensure all extracted data were accounted for. Any disagreements that arose between the reviewers were resolved through discussion.

### Data presentation

Results are reported graphically with tables when possible (Appendix 1: PRISMA checklist in the Supplemental Material). The narrative that accompanies the tables further describes the body of literature. The findings of the review are reported in two sections that were determined once the relevant sources were identified to reflect the objectives of the review. The sections are: (1) equivalent units of production and unit cost of production, and (2) fair pricing of pharmaceuticals.

## Results

### Study inclusion and exclusion

We initially identified 1,281 articles, with 1,152 excluded due to irrelevance, 76 as duplicates, and 7 for pedagogical focus (Table 3). Following screening, 46 articles were retained for analysis. A quantitative synthesis revealed that 8 articles (17.4%) explicitly addressed pharmaceutical unit production costs (cf., Table 1), only 1 empirical study (2.2%) addressed EUP practices (Guerreiro & Amaral, 2018) revealing divergence between industry methods and academic literature (cf., Table 1); and 37 articles (80.4%) examined fair pricing frameworks (cf., Table 2**)**.

Three critical research gaps emerged- in validating EUP’s role in pharmaceutical costing (see Table 1 below). First, despite 37 articles analyzing fair pricing, no direct evidence was found that pharmaceutical pricing models incorporate continuous process costing methods, including EUP. Instead, discussions centred on value-based strategies (Guerreiro & Amaral, 2018), ethical considerations (Spinello, 1992), and policy analyses (Babar, 2022).

Second, standardised methodologies and definitions for applying continuous process costing in pharmaceutical contexts remain poorly documented and understood. Third, the industry lacks state-regulated uniform costing systems and enforceable price mark-up ceilings, limiting transparency in cost determination and pricing fairness (see Table 1, and Appendices 1–4 in the Supplemental Material).

## Discussion

This study argues that accurate calculation of production costs – especially using ‘EUP' in continuous process costing – is essential for setting fair prices in the pharmaceutical industry (cf. Drury, 2021). When companies follow well-established cost accounting methods, they can show how much it truly costs to produce a drug, aligning with the auditing notion of a ‘true and fair view.'. This kind of transparency builds trust and supports pricing practices that are seen as fair. The findings support prior research on pharmaceutical fair pricing ethics (e.g. Spinello, 1992; Vogler, 2019a, 2019b) and reinforce the WHO’s emphasis on transparency as a cornerstone of fair pricing (World Health Organization [WHO], 2020).

However, while the study underscores the theoretical importance of unit cost accuracy, it reveals a critical gap: limited empirical research directly examines the unit cost formulas used in pharmaceutical production, justifying the need for further original research (e.g. Broekhof, 2002; Guerreiro & Amaral, 2018; Schreyögg et al., 2006). This is a concern because many pricing decisions rely on these cost figures. For example, even though many companies use cost-plus pricing (adding a profit margin to the production cost), there are inconsistencies in how those costs are calculated – especially when companies don’t fully account for EUP in work-in-progress scenarios. This challenges the assumption that current pricing strategies adequately reflect true production costs (Broekhof, 2002; Schreyögg et al., 2006). These gaps may be due in part to the fact that many pricing studies are done by non- accounting professionals, who may potentially overlook the technical details that affect cost accuracy.

These findings suggest that conventional cost-plus pricing models, which are often used in general management contexts, may not be the best fit for highly specialised industries like pharmaceuticals (e.g. Skinner, 1970). In such fields, where ethical standards and regulatory oversight are strong (Moon et al., 2020), policymakers are more likely to value cost data that is accurate and easy to verify. This aligns with auditing theory, which prioritises verifiability and accuracy in financial reporting (Edwards et al., 2003), and addresses concerns about information being deliberately withheld (information asymmetry) or misrepresented in pricing negotiations (World Health Organization [WHO], 2020). Importantly, the accounting profession has been largely absent from debates around drug pricing, which may be contributing to a lack of verifiable cost data. Encouraging more involvement from accounting experts could help address these issues (Modell, 2014; Van der Stede, 2011).

In practical terms, the findings support the WHO’s warning that cost-plus pricing should not be used unless it is based on consistent and reliable cost data (World Health Organization [WHO], 2020). Both companies and regulators should aim to improve how production costs are calculated and reported, including adopting standard practices like equivalent unit calculations. While other pricing models – like value-based or ethical pricing (Guerreiro & Amaral, 2018; Spinello, 1992; Suleman & Gray, 2017) – still have a role to play, they can only work effectively if the underlying cost data is accurate and verifiable – a prerequisite for fair pricing in global health contexts.

### Limitations

This scoping review shares inherent limitations of methodologies focused on conceptual clarification, such as a descriptive presentation of findings without synthesis. Nevertheless, this approach remains appropriate for defining domain-specific concepts and laying the groundwork for subsequent empirical studies. For example, the scarcity of empirical evidence on EUP (exemplified by a single study, Guerreiro & Amaral, 2018) underscores limited empirical grounding and justifies the necessity for further original research.

## Conclusions

This review demonstrates that pharmaceutical pricing frameworks lack integration with management accounting’s continuous process costing principles, particularly equivalent units of production (EUP). To address this gap, mandatory annual disclosures of regulated uniform costing methods and mark-up ceilings are urgently needed. Such measures would enhance social accountability, advance fair pricing practices, and improve medicine affordability – critical steps toward achieving UN Sustainable Development Goals (SDGs) 3 (Good Health) and 10 (Reduced Inequalities).

### Policy recommendations

In Table 4, we summarise policy recommendations or stakeholder actions that may help reinforce this study’s practical significance.

**Table 4.** Policy recommendations to strengthen EUP-based pharmaceutical pricing.

Stakeholder/Actants	Action	Outcome
**Governments**	Legislate mandatory disclosure of EUP-based unit cost formulas in pharmaceutical annual reports.	Reduces information asymmetry; enables evidence-based policymaking to cap excessive mark-ups.
	Implement uniform costing regulations (e.g. EUP benchmarks) and mark-up caps for essential medicines.	Aligns pricing with actual production costs, improving affordability and fairness.
	Establish public oversight bodies to audit costing practices and enforce transparency standards.	Strengthens accountability and public trust in pricing systems.
**Pharmaceutical Companies**	Adopt EUP-based costing methods to standardise cost allocation across continuous production lines.	Enhances internal cost efficiency and justifies pricing decisions to regulators/public.
	Publish granular cost breakdowns (e.g. raw materials, labour, overhead) linked to EUP metrics.	Builds trust with stakeholders and supports claims of ‘fair pricing' in line with SDGs.
	Collaborate with regulators to pilot fair pricing models tied to SDG 3 (health equity).	Balances profit motives with social accountability, improving brand reputation.
**Regulatory Bodies**	Develop EUP accounting guidelines tailored to pharmaceutical manufacturing (e.g. work-in-progress valuation).	Creates industry-wide consistency in cost reporting and reduces arbitrariness in pricing.
	Require EUP-based cost disclosures as a precondition for drug approval or tenders.	Links market access to transparency, discouraging exploitative pricing.
	Penalize firms for non-compliance (e.g. fines, exclusion from subsidies) to deter opacity.	Deters unethical costing practices and incentivizes adherence to standards.
**International Organizations (WHO, UN)**	Advocate for global adoption of EUP-based costing frameworks through SDG-aligned partnerships.	Harmonizes pricing norms across countries, reducing cross-border price disparities.
	Publish annual rankings of nations based on pharmaceutical pricing transparency and fairness.	Encourages competition among governments to meet SDG 3 (health) and SDG 10 (reduced inequalities) targets.
**Civil Society & NGOs**	Campaign for public access to drug cost data and challenge opaque pricing via litigation.	Empowers patients and healthcare providers to demand accountability from firms and governments.
	Educate communities on the link between production costs and medicine affordability.	Strengthens public pressure for pricing reforms and equitable access.
**Healthcare Providers**	Prioritize procurement of medicines from firms complying with EUP-based cost disclosures.	Rewards transparent manufacturers, creating market incentives for ethical pricing.
	Partner with regulators to flag discrepancies between reported costs and market prices.	Provides real-world data to refine EUP models and address loopholes.

### Future research

Further work is needed to quantify EUP’s impact on fair pricing across diverse regulatory contexts and explore digital tools for real-time cost tracking. Future research could address: (1) What organisational, technological, and regulatory factors influence EUP adoption in pharmaceutical costing? (2) How does EUP integration improve cost accuracy and decision-making compared to existing methods (e.g. batch costing)? Data could be sourced from: (1) Case studies – embedded researchers in auditing teams conducting interviews and analysing costing data. (2) Expert surveys – targeting professionals (e.g. cost accountants, supply chain managers) to identify challenges and success factors.

## Supplementary Material

Supplemental Material

## Data Availability

All data generated or analysed during this study are included in this published article [and its Supplemental Material].

## Footnotes

1 Actor-Network Theory (ANT) (Latour, 1987) examines how both human and non-human ‘actants’ (e.g., drugs, patents, regulations) collectively shape outcomes through dynamic networks. In pharmaceutical pricing, ANT highlights: a. Actants: – Human: Pharma companies, regulators, insurers, patients, lobbyists. – Non-human: Patents (granting exclusivity), drug efficacy data (justifying cost), manufacturing technologies (affecting production expenses), and pricing algorithms. b. Interactions: Pricing emerges from negotiations among actants. For example, patents (non-human) enable monopolistic pricing, while regulatory agencies (human) may constrain it. Insurance companies (human) negotiate reimbursements, influenced by clinical trial data (non-human). c. Translation: Actors align interests to stabilize the network. Pharma firms might enlist doctors (via marketing) or patient groups (via advocacy) to justify high prices, while generic drug entry (post-patent expiration) disrupts the network, lowering prices. d. Agency of non-humans: A drug's chemical complexity or production challenges (non-human) can elevate costs, while public outcry (human) may pressure policymakers to regulate prices.

2 Van der Stede (2011) calls for ‘management accounting to be turned inside-out.’

## References

[CIT0001] Amaral, J. V., & Guerreiro, R. (2019). Factors explaining a cost-based pricing essence. *Journal of Business and Industrial Marketing*, *34*(8), 1850–1865. 10.1108/JBIM-12-2018-0373

[CIT0002] Andersson, F., & McMenamin, P. (1992). International price comparisons of pharmaceuticals – A review of methodological issues (*London and Washington). Battelle Medical Technology and Policy Research Centre (MEDTAP)*.

[CIT0003] Arksey, H., & O’Malley, L. (2005). Scoping studies: Towards a methodological framework. *International Journal of Social Research Methodology*, *8*(1), 19–32. 10.1080/1364557032000119616

[CIT0004] Babar, Z. U. D. (2015). *Pharmaceutical prices in the 21st century (Ed)*. Adis Cham. 10.1007/978-3-319-12169-7

[CIT0005] Babar, Z. U. D. (2022). Forming a medicines pricing policy for low and middle-income countries (LMICs): The case for Pakistan. *Journal of Pharmaceutical Policy and Practice*, *15*(1), 9. 10.1186/s40545-022-00413-3 35209945 PMC8867617

[CIT0006] Babar, Z. U. D., Ibrahim, M. I. M., Singh, H., Bukhari, N. I., & Creese, A. (2007). Evaluating drug prices, availability, affordability, and price components: Implications for access to drugs in Malaysia. *PLoS Medicine*, *4*(3), e82. 10.1371/journal.pmed.0040082 17388660 PMC1831730

[CIT0007] Basu, P., Joglekar, G., Rai, S., Suresh, P., & Vernon, J. (2008). Analysis of manufacturing costs in pharmaceutical companies. *Journal of Pharmaceutical Innovation*, *3*(1), 30–40. 10.1007/s12247-008-9024-4

[CIT0008] Bendicksen, L., Rome, B. N., Avorn, J., & Kesselheim, A. S. (2021). Pursuing value-based prices for drugs: A comprehensive comparison of state prescription drug–pricing boards. *The Milbank Quarterly*, *99*(4), 1162–1197. 10.1111/1468-0009.12533 34375015 PMC8718587

[CIT0009] Berlak, J., & Götz, T. (2021). Concept for the urban production of pharmaceuticals to compensate for local shortages. *Digital Manufacturing Technology*, *1*(1), 46–59. 10.37256/dmt.112021910

[CIT0010] Bischof, R. O. (1996). Pharmaceuticals in the era of cost-containment. *Hospital Practice*, *31*(*12*), 77–84. 10.1080/21548331.1996.11443392 8969681

[CIT0011] Broekhof, M. (2002). *Transparency in the pharmaceutical industry – A cost accounting approach to the prices of drugs*. University of Groningen.

[CIT0012] Cohn, J. (2016). The drug price controversy nobody notices. *The Milbank Quarterly*, *94*(2), 260–263. 10.1111/1468-0009.12193 27265558 PMC4911722

[CIT0013] Daudt, H. M., van Mossel, C., & Scott, S. (2013). Enhancing the scoping study methodology: A large, inter-professional team’s experience with Arksey and O’Malley’s framework. *BMC Medical Research Methodology*, *13*(1), 48. 10.1186/1471-2288-13-48 23522333 PMC3614526

[CIT0014] Davenport, C., Norkus, J., & Simonetto, M. (2012). Capturing the value of pricing analytics. In M. N. Smith & L. S. Gordon (Eds.), *Visionary pricing: Reflections and advances in honor of Dan Nimer* (pp. 3–20). Emerald Group Publishing Limited. 10.1108/S1069-0964(2012)0000019019

[CIT0015] Drury, C. (2021). *Cost and management accounting 11*. CENTAGE.

[CIT0016] Drury, C., & Tayles, M. (1994). Product costing in UK manufacturing organisations. *The European Accounting Review*, *3*(3), 443–469. 10.1080/09638189400000031

[CIT0017] Edwards, J. R., Boyns, T., & Matthews, M. (2003). Costing, pricing and politics in the British steel industry, 1918-1967. *Management Accounting Research*, *14*(1), 25–49. 10.1016/S1044-5005(02)00033-1

[CIT0018] Guerreiro, R., & Amaral, J. V. (2018). Cost-based price and value-based price: Are they conflicting approaches? *Journal of Business and Industrial Marketing*, *33*(3), 390–404. 10.1108/JBIM-04-2016-0085

[CIT0019] Hill, A., Barber, M., & Gotham, D. (2018). Estimated costs of production and potential prices for the WHO essential medicines list. *BMJ Global Health*, *3*(1), e000571. 10.1136/bmjgh-2017-000571 PMC5859811 29564159

[CIT0020] Landis, J. R., & Koch, G. G. (1977). An application of hierarchical Kappa-type statistics in the assessment of majority agreement among multiple observers. *Biometrics*, *33*(*2*), 363. 10.2307/2529786 884196

[CIT0021] Latour, B. (1987). *Science in action*. Harvard University Press. 10.1177/016224399001500407

[CIT0022] Levac, D., Colquhoun, H., & O’Brien, K. K. (2010). Scoping studies: Advancing the methodology. *Implementation Science*, *5*(69), 1–9. 10.1186/1748-5908-5-69 20854677 PMC2954944

[CIT0023] Modell, S. (2014). The societal relevance of management accounting: An introduction to the special issue. *Accounting and Business Research*, *44*(2), 88–103. 10.1080/00014788.2014.882741

[CIT0024] Moon, S., Mariat, S., Kamae, I., & Pedersen, H. B. (2020). Defining the concept of fair pricing for medicines. *BMJ*, *368*, l4726. 10.1136/bmj.l4726 31932334

[CIT0025] Peters, M. D. J., Godfrey, C. M., Khalil, H., McInerney, P., Parker, D., & Soares, C. B. (2015). Guidance for conducting systematic scoping reviews. *International Journal of Evidence-Based Healthcare*, *13*(*3*), 141–146. 10.1097/XEB.0000000000000050 26134548

[CIT0026] Peters, M. D. J., Godfrey, C. M., Khalil, H., McInerney, P., Parker, D., & Soares, C. B. (2015). Guidance for conducting systematic scoping reviews. *International Journal of Evidence-Based Healthcare*, *13*(3), 141–146. 10.1097/XEB.0000000000000050 26134548

[CIT0027] Pinheiro, E., Vasan, A., Kim, J. Y., Lee, E., Guimier, J. M., & Perriens, J. (2006). Examining the production costs of antiretroviral drugs. *Aids (london, England)*, *20*(13), 1745–1752. 10.1097/01.aids.0000242821.67001.65 16931939

[CIT0028] Scherer, F. (2000). The pharmaceutical industry. *Handbook of Health Economics*, *1*, 1297–1336. 10.1016/S1574-0064(00)80038-4

[CIT0029] Schreyögg, J., Tiemann, O., & Busse, R. (2006). Cost accounting to determine prices: How well do prices reflect costs in the German DRG-system? *Health Care Management Science*, *9*(*3*), 269–279. 10.1007/s10729-006-9094-0 17016933

[CIT0030] Skinner, R. (1970). The determination of selling prices. *Journal of Industrial Economics*, *18*(3), 201–217. 10.2307/2097610

[CIT0031] Spinello, R. A. (1992). Ethics, pricing and the pharmaceutical industry. *Journal of Business Ethics*, *11*(8), 617–626. 10.1007/BF00872273

[CIT0032] Suleman, F., & Gray, A. (2017). Pharmaceutical policy in South Africa. In Z.-U.-D. Babar (Ed.), *Pharmaceutical policy in countries with developing healthcare systems*. Adis Cham. 10.1007/978-3-319-51673-8_14

[CIT0033] Syversen, I. D., Schulman, K., Kesselheim, A. S., & Feldman, W. B. (2024). A comparative analysis of international drug price negotiation frameworks: An interview study of key stakeholders. *Milbank Quarterly*, *102*(4), 1004–1031. 10.1111/1468-0009.12714 39289915 PMC11654763

[CIT0034] Towse, A., Hernandez-Villafuerte, K., & Shaw, B. (2018). *A critique of the paper “The estimated costs of production and potential prices for the World Health Organization Essential Medicines List” Consulting Report*. www.ohe.org.

[CIT0035] Van der Stede, W. A. (2011). Management accounting research in the wake of the crisis: Some reflections. *European Accounting Review*, *20*(4), 605–623. 10.1080/09638180.2011.627678

[CIT0036] Vogler, S. (2018). Access to high-cost medicines in Europe. In Z.-U.-D. Babar (Ed.), *Equitable access to high-cost pharmaceuticals* (pp. 143–164). Elsevier. 10.1016/b978-0-12-811945-7.00010-5

[CIT0037] Vogler, S. (2019a). Assessment of external price referencing and alternative policies. In S. Vogler (Ed.), *Medicine price surveys, analyses and comparisons* (pp. 369–419). Academic Press. 10.1016/B978-0-12-813166-4.00019-X

[CIT0038] Vogler, S. (2019b). Fair prices for medicines? Exploring competent authorities’ and public payers’ preferences on pharmaceutical policies. *Empirica*, *46*, 1–27. 10.1007/S10663-019-09446-5

[CIT0039] Vogler, S., Kilpatrick, K., & Babar, Z. (2015). Analysis of medicine prices in New Zealand and 16 European countries. *Value in Health: The Journal of the International Society for Pharmacoeconomics and Outcomes Research*, *18*(4), 484–492. 10.1016/j.jval.2015.01.003 26091603

[CIT0040] Vogler, S., & Martikainen, J. E. (2015). Pharmaceutical pricing in Europe. In Z.-U.-D. Babar (Ed.), *Pharmaceutical prices in the 21st century*. Adis Cham. 10.1007/978-3-319-12169-7_19

[CIT0041] Vogler, S., Vitry, A., & Babar, ZUD. (2016). Cancer drugs in 16 European countries, Australia, and New Zealand: A cross-country price comparison study. *The Lancet Oncology*, *17*(*1*), 39–47. 10.1016/S1470-2045(15)00449-0 26670089

[CIT0042] Walley, T. (2007). Fair pricing for medicines in the UK. *Expert Review of Pharmacoeconomics & Outcomes Research*, *7*(3), 207–209. 10.1586/14737167.7.3.207 20528306

[CIT0043] Wilensky, G. R. (2016). Prescription drug pricing is not just an election issue. *The Milbank Quarterly*, *94*(4), 712–715. 10.1111/1468-0009.12223 27995706 PMC5192878

[CIT0044] World Health Organization. (2020). WHO guideline on country pharmaceutical pricing policies. https://www.who.int/publications/i/item/9789240011878 25473702

[CIT0045] Zawawi, N., & Hoque, Z. (2008). Research in management accounting innovations: An overview of its development. *Qualitative Research in Accounting & Management*, *7*(4), 505–568. 10.1108/11766091011094554

