# Supplementary material for: Impact of equivalent units of production on state-controlled unit cost calculation for fair pricing of pharmaceuticals: a scoping review
Source: J Pharm Policy Pract. 2025 Oct 23;18(1):2564820. doi: 10.1080/20523211.2025.2564820 (PMC12551011; doi:10.1080/20523211.2025.2564820)
Supplement: Supplemental Material [file JPPP_A_2564820_SM8735.pdf]

IMPACT OF EQUIVALENT UNITS OF PRODUCTION ON FAIR PRICING OF PHARMACEUTICALS: A SCOPING REVIEW

Search results and source selection and inclusion process- PRISMA checklist

17/11/23

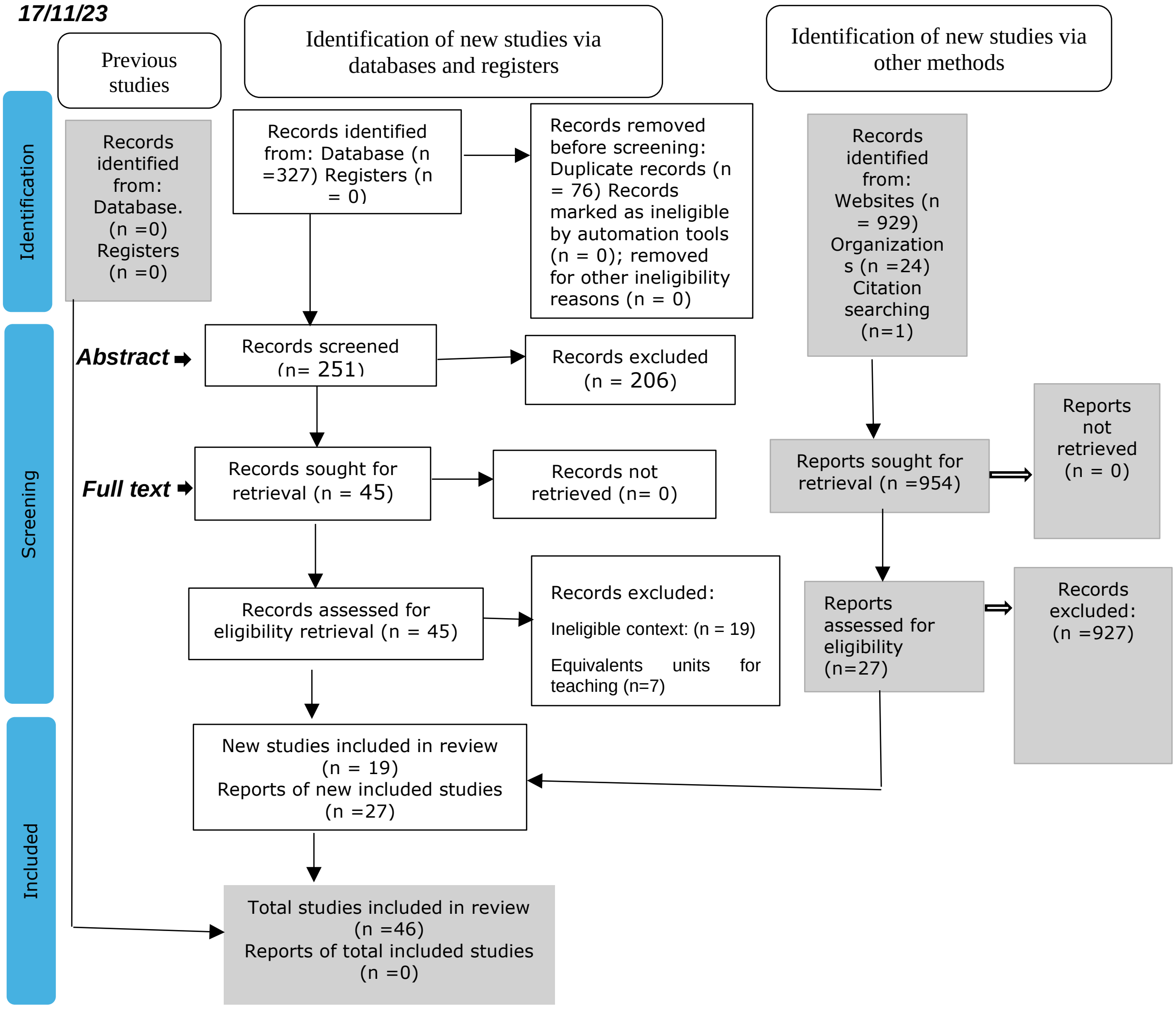

Moher D, Liberati A, Tetzlaff J, Altman DG; the PRISMA Group. Preferred Reporting Items for Systematic Reviews and Meta-Analyses: The PRISMA Statement. PLoS Med. 2009;6(7): e1000097

Appendix 2: Data extraction instrument

Reviewer: ____

Date: ____

Information to be extracted	
Author (s)	
Year of publication	
Citation information	
Origin (country)	
Type of literature (research [include if thesis] or policy document)	
Aim/purpose	
Design/methods	
Study population	
Concept	
How concept was defined	
Industry (pharmaceuticals, manufacturing etc.)	

Appendix 3: Characteristics of excluded studies

Reason for exclusion: pricing reports (n=3)

Abbott, T. (2002). Committee on Aging report Earning a Failing Grade: A Report Card on 1992 Drug Manufacturer Price Inflation. 2002; Available from: https://www.semanticscholar.org/paper/0656b5d94bc71ee339db42d788a92b4343c1488d

Kheirandish, M. (2020). Do WHO pharmaceutical pricing guidelines help low-and middle-in-come countries to price medicines rationally?. *Eastern Mediterranean Health Journal*, *26*(4), 372-373. https://doi.org/10.26719/2020.26.4.372

Koduah A, Baatiema L, Chavez ACD, Danso-Appiah A, Agyepong IA, King N, et al. (2021). Implementation of Medicines Pricing Policies in Sub-Saharan Africa: Systematic Review. https://doi.org/10.21203/rs.3.rs-1107173/v1

Reason for exclusion: regulations (n=16)

Abbott III, T. A. (1995). Price regulation in the pharmaceutical industry: Prescription or placebo?. *Journal of Health Economics*, *14*(5), 551-565. https://doi.org/10.1016/0167-6296(95)00022-4

Bangalee, V., & Suleman, F. (2015). Evaluating the effect of a proposed logistics fee cap on pharmaceuticals in South Africa-a pre and post analysis. *BMC health services research*, *15*, 1-12. https://doi.org/10.1186/s12913-015-1184-6

Bangalee, V., & Suleman, F. (2016). Towards a transparent pricing system in South Africa in pharmaceutical logistics fees. *South African health review*, *2016*(1), 221-231. https://hdl.handle.net/10520/EJC189306

Colak B. (2014). Generic Competition and Price Regulation in the European Union Pharmaceutical Market: The Case of Cardiovascular Medicines. USF Tampa Graduate Theses and Dissertations. https://digitalcommons.usf.edu/etd/5000

Geng, D., & Saggi, K. (2020). Optimal price regulations in international pharmaceutical markets with generic competition. *Journal of Health Economics*, *71*, 102315. https://doi.org/10.1016/j.jhealeco.2020.102315

Grabowski, H. G., Vernon, J. M., & Thomas, L. G. (1978). Estimating the effects of regulation on

innovation: an international comparative analysis of the pharmaceutical industry. *The Journal of Law and Economics*, *21*(1), 133-163. https://doi.org/10.3389/fmed.2023.1264021
Grabowski, H. G., & Vernon, J. M. (1992). Brand loyalty, entry, and price competition in pharmaceuticals after the 1984 Drug Act. *The journal of law and economics*, *35*(2), 331-350. https://doi.org/10.1016/j.healthpol.2023.104818
Gray A. Medicine pricing interventions - the South African experience. 2009. Gray, A. L. (2009). Medicine pricing interventions--the South African experience. *Southern Med Review*, *2*(2). https://www.semanticscholar.org/paper/21a17d05d3e29fe8af259112e54225fda07a3f52
Kaiser, U., Mendez, S. J., Rønde, T., & Ullrich, H. (2014). Regulation of pharmaceutical prices: evidence from a reference price reform in Denmark. *Journal of health economics*, *36*, 174-187. https://doi.org/10.1016/j.jhealeco.2014.04.003
Kohler, J. C., Mitsakakis, N., Saadat, F., Byng, D., & Martinez, M. G. (2015). Does pharmaceutical pricing transparency matter? Examining Brazil's public procurement system. *Globalization and health*, *11*, 1-13. https://doi.org/10.1186/s12992-015-0118-8
Prada, S. I., Soto, V. E., Andia, T. S., Vaca, C. P., Morales, Á. A., Márquez, S. R., & Gaviria, A. (2018). Higher pharmaceutical public expenditure after direct price control: improved access or induced demand? The Colombian case. *Cost Effectiveness and Resource Allocation*, *16*, 1-8. https://doi.org/10.1186/s12962-018-0092-0
Puig-Junoy, J. (2010). Impact of European pharmaceutical price regulation on generic price competition: a review. *Pharmacoeconomics*, *28*, 649-663. https://doi.org/10.2165/11535360-000000000-00000
Bardey, David and Harker, Arturo and Zuluaga, Daniela, Price Cap Regulation in the Colombian Pharmaceutical Market: An Impact Evaluation (March 10, 2021). Documento CEDE No. 9, Available at SSRN: https://ssrn.com/abstract=3805791 or http://dx.doi.org/10.2139/ssrn.3805791
Temin, P. (1979). Technology, regulation, and market structure in the modern pharmaceutical industry. *The Bell Journal of Economics*, 429-446. https://doi.org/10.2307/3003345
Vogler, S., Habimana, K., & Arts, D. (2014). Does deregulation in community pharmacy impact accessibility of medicines, quality of pharmacy services and costs? Evidence from nine European countries. *Health policy*, *117*(3), 311-327. https://doi.org/10.1016/j.healthpol.2014.06.001

Reason for exclusion: pharmaeconomics studies (n-2)

Abbott III, T. A. (1995). Price regulation in the pharmaceutical industry: Prescription or placebo?. *Journal of Health Economics*, *14*(5), 551-565. https://doi.org/10.1016/0167-6296(95)00022-4
Acosta, A., Ciapponi, A., Aaserud, M., Vietto, V., Austvoll-Dahlgren, A., Kösters, J. P., ... & Oxman, A. D. (2014). Pharmaceutical policies: effects of reference pricing, other pricing, and purchasing policies. *Cochrane Database of Systematic Reviews*, (10). https://doi.org/10.1002/14651858.CD005979.pub2

Reason for exclusion: pricing policies studies (n=18)

Acosta, A., Ciapponi, A., Aaserud, M., Vietto, V., Austvoll-Dahlgren, A., Kösters, J. P., ... & Oxman, A. D. (2014). Pharmaceutical policies: effects of reference pricing, other pricing, and purchasing policies. *Cochrane Database of Systematic Reviews*, (10). https://doi.org/10.1002/14651858.CD005979.pub2
Anggriani, Y., Ibrahim, M. I. M., Suryawati, S., & Shafie, A. A. (2013). The impact of Indonesian generic medicine pricing policy on medicine prices. *Journal of Generic Medicines*, *10*(3-4), 219-229. https://doi.org/10.1177/1741134314553605
Anggriani, Y. (2018). The Role of Medicine Pricing Policy for Improving the Affordability of Medicines. *JURNAL ILMU KEFARMASIAN INDONESIA*, *16*(2), 172-181.

https://doi.org/10.35814/jifi.v16i2.550
Carone, Giuseppe and Schwierz, Christoph and Xavier, Ana, Cost-Containment Policies in Public Pharmaceutical Spending in the EU (September 14, 2012). Available at SSRN: https://ssrn.com/abstract=2161803 or http://dx.doi.org/10.2139/ssrn.2161803
Fulda TR, Wertheimer A. Handbook of Pharmaceutical Public Policy. 2007. https://www.semanticscholar.org/paper/81458113438167d08da304a2f4c79b355d68adb8
Kong, Y. (2004). The price premium of generic to brand-names and pharmaceutical price index. *Applied Economics*, *36*(7), 731-737. https://doi.org/10.1080/0003684042000222106
Leopold, C., Zhang, F., Mantel-Teeuwisse, A. K., Vogler, S., Valkova, S., Ross-Degnan, D., & Wagner, A. K. (2014). Impact of pharmaceutical policy interventions on utilization of antipsychotic medicines in Finland and Portugal in times of economic recession: interrupted time series analyses. *International Journal for Equity in Health*, *13*(1), 1-9. https://doi.org/10.1186/1475-9276-13-53
Liozu, S.M. and Hinterhuber, A. (2022), "Pricing and CEOs: why top executives need to get involved", *Journal of Business Strategy*, Vol. 43 No. 5, pp. 283-290. https://doi.org/10.1108/JBS-02-2021-0024
Maniadakis, N., Kourlaba, G., Shen, J., & Holtorf, A. (2017). Comprehensive taxonomy and worldwide trends in pharmaceutical policies in relation to country income status. *BMC Health Services Research*, *17*, 1-17. https://doi.org/10.1186/s12913-017-2304-2
Nguyen, T. A., Knight, R., Roughead, E. E., Brooks, G., & Mant, A. (2015). Policy options for pharmaceutical pricing and purchasing: issues for low-and middle-income countries. *Health Policy and Planning*, *30*(2), 267-280. https://doi.org/10.1093/heapol/czt105
Rida, N. A., & Ibrahim, M. I. M. (2018). Medicines pricing policy and strategies in developing countries: A review. *Social and administrative aspects of pharmacy in low-and middle-income countries*, 111-128. . https://doi.org/10.1016/B978-0-12-811228-1.00007-8
Rudisill, C., Vandoros, S., & Antoun, J. G. (2014). Pharmaceutical policy reform in the Russian Federation. *Journal of health politics, policy and law*, *39*(3), 691-705. https://doi.org/10.1215/03616878-2682659
Suleman, F., Gray, A. (2017). Pharmaceutical Policy in South Africa. In: Babar, ZUD. (eds) Pharmaceutical Policy in Countries with Developing Healthcare Systems. Adis, Cham. https://doi.org/10.1007/978-3-319-51673-8_14
Vogler, S., Zimmermann, N., & Habimana, K. (2016). Stakeholder preferences about policy objectives and measures of pharmaceutical pricing and reimbursement. *Health Policy and Technology*, *5*(3), 213-225. https://doi.org/10.1016/j.hlpt.2016.03.009
Vogler S, Zimmermann N, Habimana K. Study of the policy mix for the reimbursement of Medicinal products. Proposal for a best practice-based approach based on stakeholder assessment. 2014. https://www.semanticscholar.org/paper/d5da3d9932a116bb2640249e8fd5f8d17a517158
Vogler, S., Zimmermann, N., & Habl, C. (2013). Understanding the components of pharmaceutical expenditure-overview of pharmaceutical policies influencing expenditure across European countries. *GaBi J*, *2*(3), 178-187. https://www.semanticscholar.org/paper/d26757a8bd7a433e643eea0bdd4214e1e45d29b3
Vogler, S., Zimmermann, N. Joncheere,K.de (2016). Policy interventions related to medicines: Survey of measures taken in European countries during 2010–2015, Health Policy,120(12),2016. https://doi.org/10.1016/j.healthpol..09.006.
Vogler, S., Zimmermann, N., Leopold, C., Habl, C., & Mazag, J. (2013). Discounts and rebates granted for medicines for hospital use in five European countries. *The Open*

Pharmacoeconomics & Health Economics Journal, *5*(1). http://dx.doi.org/10.2174/1876824501305010001

Reason for exclusion: primary care studies(n=9)

Anell, A., & Glenngård, A. H. (2014). The use of outcome and process indicators to incentivize. integrated care for frail older people: a case study of primary care services in Sweden. *International journal of integrated care*, *14*. https://doi.org/10.5334/ijic.1680

Anell A. Performance management and audit & feedback to support learning and innovation: Theoretical review and implications for Swedish primary care. 2019; Available from: https://www.semanticscholar.org/paper/640cea4cd6b12fde98f1bdeec997b3604526149b

De Bekker, P., Vink, M., Donker, M., Sterkenburg, P., & van der Hijden, E. (2020). GPs perspective on the usability of the Dutch Primary Care Practice Report: qualitative interpretative approach. https://doi.org/10.21203/rs.3.rs-19290/v1

Ellegård, L. M. (2020). Effects of pay-for-performance on prescription of hypertension drugs. among public and private primary care providers in Sweden. *International journal of health economics and management*, *20*(3), 215-228. https://doi.org/10.1007/s10754-020-09278-y

Glenngård, A. H. (2016). Experiences of introducing a quasi-market in Swedish primary care: Fulfilment of overall objectives and assessment of provider activities. *Scandinavian Journal of Public Administration*, *21*(1), 71–86. Retrieved from https://publicera.kb.se/sjpa/article/view/14980

Glenngård, A. H. (2013). Is patient satisfaction in primary care dependent on structural and organizational characteristics among providers? Findings based on data from the national patient survey in Sweden. *Health Economics, Policy and Law*, *8*(3), 317-333. https://doi.org/10.1017/S1744133112000333

Glenngård, A. H. (2019). Pursuing the objectives of support to providers and external accountability through enabling controls-A study of governance models in Swedish primary care. *BMC Health Services Research*, *19*, 1-14. https://doi.org/10.1186/s12913-019-3945-0

Glenngård, A. H. (2021). What matters for patients' experiences with primary care? A study of variation in patient reported experience measures with regard to structural and organisational characteristics of primary care centres in a Swedish region. *Nordic Journal of Health Economics*. https://doi.org/10.5617/njhe.8030

Vogler, S., Zimmermann, N., Leopold, C., & de Joncheere, K. (2011). Pharmaceutical policies in European countries in response to the global financial crisis. *Southern med review*, *4*(2), 69. https://doi.org/10.5655/smr.v4i2.1004

Reason for exclusion: reimbursement studies (n=6)

Atikeler, K., & Ozcelikay, G. (2015). Comparison of pharmaceutical pricing and reimbursement systems in turkey and certain other EU countries. *Value in Health*, *18*(7), A572. https://doi.org/10.1016/j.jval.2015.09.1889

Vogler, S., Habl, C., Bogut, M., & Vončina, L. (2011). Comparing pharmaceutical pricing and reimbursement policies in Croatia to the European Union Member States. *Croatian medical journal*, *52*(2), 183-197. https://doi.org/10.3325/cmj.2011.52.183

Vogler, S., Leopold, C., Zimmermann, N., Habl, C., & de Joncheere, K. (2014). The pharmaceutical pricing and reimbursement information (PPRI) initiative—experiences from engaging with pharmaceutical policy makers. *Health Policy and Technology*, *3*(2), 139-148. https://doi.org/10.1016/j.hlpt.2014.01.001

Vogler, S., Paris, V., Ferrario, A., Wirtz, V. J., de Joncheere, K., Schneider, P., ... & Babar, Z. U.

D. (2017). How can pricing and reimbursement policies improve affordable access to medicines? Lessons learned from European countries. Applied health economics and health policy, 15, 307-321. https://doi.org/10.1007/s40258-016-0300-z
Vogler, S., Zimmermann, N., Ferrario, A., Wirtz, V. J., & Babar, Z. U. D. (2015). Challenges and opportunities for pharmaceutical pricing and reimbursement policies. *Journal of Pharmaceutical Policy and Practice*, *8*(1), 1-3. https://doi.org/10.1186/2052-3211-8-S1-E1
Vogler, S. (2012). The impact of pharmaceutical pricing and reimbursement policies on generics uptake: implementation of policy options on generics in 29 European countries–an overview. *Generics and Bisimilars Initiative Journal*, *1*(2), 44-51. Available from: https://www.semanticscholar.org/paper/153f5bdab5dc462f778e5546824467ee4ac65dde

Reason for exclusion: impact of competition on prices studies (n-5)

Bangalee, V., & Suleman, F. (2016). Has the increase in the availability of generic drugs lowered the price of cardiovascular drugs in South Africa?. *health sa gesondheid*, *21*(1), 60-66. https://hdl.handle.net/10520/EJC186732
Vogler, S., Zimmermann, N., Habl, C., & Mazag, J. (2013). The role of discounts and loss leaders in medicine procurement in Austrian hospitals-a primary survey of official and actual medicine prices. Cost effectiveness and resource allocation, 11(1), 1-10. https://doi.org/10.1186/1478-7547-11-15
Vogler, S., Zimmermann, N., Habl, C., Piessnegger, J., & Bucsics, A. (2012). Discounts and rebates granted to public payers for medicines in European countries. *Southern med review*, *5*(1), 38-46. Epub 2012 Jul 23. PMID: 23093898; PMCID: PMC3471187. Available from: https://www.semanticscholar.org/paper/0c6392a3f632a059d86b4a9134a8ee5931c1fffa
Vogler, Sabine, et al. "Availability and procurement conditions of originator and generic medicines in hospitals--an exploratory study in five medium-sized European countries." *Generics and Biosimilars Initiative Journal*, vol. 3, no. 4, Dec. 2014, pp. 168+. *Gale Academic One File*. link.gale.com/apps/doc/A513529245/AONE?u=anon~eeaf33e0&sid=googleScholar&xid=3529fe00. Accessed 21 Nov. 2023. Available from: https://www.semanticscholar.org/paper/a5bc95928a0204cbf510390a56de10456c846c5a
Vogler, Sabine, et al. "Availability and procurement conditions of originator and generic medicines in hospitals--an exploratory study in five medium-sized European countries." *Generics and Biosimilars Initiative Journal*, vol. 3, no. 4, Dec. 2014, pp. 168+. *Gale Academic One File*. link.gale.com/apps/doc/A513529245/AONE?u=anon~5532b9b4&sid=googleScholar&xid=3f771934 . Accessed 21 Nov. 2023. Available from: https://www.semanticscholar.org/paper/a5bc95928a0204cbf510390a56de10456c846c5a

Reason for exclusion: comparative studies (n=24)

Danzon, P. M., & Kim, J. D. (2002). *The life cycle of pharmaceuticals: a cross-national perspective*. London: Office of Health Economics; Available from: https://www.semanticscholar.org/paper/d193516ec85067f2243fdd6260d8c36027ab6d70
Davenport, C., Norkus, J. and Simonetto, M. (2012), "Capturing the Value of Pricing Analytics", Smith, G.E. (Ed.) *Visionary Pricing: Reflections and Advances in Honor of Dan Nimer* (*Advances in Business Marketing and Purchasing, Vol. 19*), Emerald Group Publishing Limited, Leeds, pp. 299-333. https://doi.org/10.1108/S1069-0964(2012)0000019019
Day, C., Groenewald, P., Laubscher, R., van Schaik, N., & Bradshaw, D. (2014). Monitoring of

non-communicable diseases such as hypertension in South Africa: Challenges for the post-2015 global development agenda. *South African medical journal*, *104*(10), 680-687. Available from: https://www.semanticscholar.org/paper/f990e5c831e8e7088bd5e161586999236b4ab5fe

Ellison, S. F., Cockburn, I., Griliches, Z., & Hausman, J. (1997). Characteristics of demand for pharmaceutical products: an examination of four cephalosporins. *The RAND journal of Economics*, 426-446. https://doi.org/10.2307/2556023

Ewen, M., Kaplan, W., Gedif, T., Justin-Temu, M., Vialle-Valentin, C., Mirza, Z., ... & Laing, R. (2017). Prices and availability of locally produced and imported medicines in Ethiopia and Tanzania. *Journal of pharmaceutical policy and practice*, *10*(1), 1-9. https://doi.org/10.1186/s40545-016-0095-1

Frank, R. G., & Salkever, D. S. (1997). Generic entry and the pricing of pharmaceuticals. *Journal of Economics & Management Strategy*, *6*(1), 75-90. https://doi.org/10.1111/j.1430-9134.1997.00075.x

Gerber, D. (2006). *The Economic Significance of the Pharmaceutical Wholesaler in South Africa's Health Care Industry* (Doctoral dissertation, University of South Africa). Available from: https://www.semanticscholar.org/paper/4724aac4f0a3d4dc5d138c3210957502a43d792e

Godman, B., Hill, A., Simoens, S., Kurdi, A., Gulbinovič , J., Martin, A., Timoney, A., Gotham, D., Wale, J., Bochenek , T., Rothe, C., Hoxha, I., Malaj, A., Hierländer, C., Sauermann, R., Hamelinck, W., Mitkova, Z., Petrova, G., Laius, O., ... Haycox, A. (2019). Pricing of oral generic cancer medicines in 25 European countries; findings and implications. *Generics and Biosimilars Initiative journal*, *8*(2), 49-70. Advance online publication. https://doi.org/10.5639/gabij.2019.0802.007

Godman, B., Kwon, H. Y., Sović Brkičić, L., Bogut, M., Sršen, M., Tabain, T., & de Bruyn, W. (2015). Pharmaceutical pricing in Croatia: a comparison of ordinances in 2013 versus 2009 and their potential savings to provide future guidance. *Generics and Biosimilars Initiative Journal*, *4*(2), 79-89. https://doi.org/10.5639/gabij.2015.0402.017

Gray, A. (2014). Medicines shortages—unpicking the evidence from a year in South Africa. *The Australasian medical journal*, *7*(5), 208. https://doi.org/10.4066/AMJ.2014.1932

Griliches Z, Cockburn I. Generics and New Goods in Pharmaceutical Price Indexes. 1993; Available from: https://www.semanticscholar.org/paper/0e0bca87d992d2e8d5af79702c7be2db1c903de1

Kwon, H. Y., Kim, H., & Godman, B. (2018). Availability and affordability of drugs with a conditional approval by the European Medicines Agency; comparison of Korea with other countries and the implications. *Frontiers in pharmacology*, *9*, 938. https://doi.org/10.3389/fphar.2018.00938

Petrou P, Vandoros S. Pharmaceutical price comparisons across the European Union and Relative affordability in Cyprus. 2016;5(4):356. https://doi.org/10.1016/j.hlpt.2016.07.009.

Reiffen, D., & Ward, M. R. (2005). Generic drug industry dynamics. *Review of Economics and statistics*, *87*(1), 37-49. https://doi.org/10.1162/0034653053327694

Rohra, D. K., Abuomar, O. A., Cahusac, P. M., Dangol, A., & Ranasinghe, P. (2020). Comparative analysis of prescription drug prices in South Asian Association for Regional Cooperation Countries. *Value in Health Regional Issues*, *21*, 113-119. https://doi.org/10.1016/j.vhri.2019.09.004

Scherer, F. M. (1993). Pricing, profits, and technological progress in the pharmaceutical industry. *Journal of Economic Perspectives*, *7*(3), 97-115. https://doi.org/10.1257/jep.7.3.97

Simoens PDS. A review of generic medicine pricing in Europe. 2012. *Generics and Biosimilars Initiative Journal*,1(1). Available from: https://www.semanticscholar.org/paper/faf2fb5b0ff30dcb4b67149022c87557be68ccdf

Vogler S, Kilpatrick K, Babar Z. Analysis of Medicine Prices in New Zealand and 16 European Countries. 2015;18 4:492. https://doi.org/10.1016/j.jval.2015.01.003.

Vogler S, Lepuschütz L, Schneider P. Pharmaceutical distribution remuneration in Europe. 2015;8: P23. Vogler, S., Lepuschütz, L. & Schneider, P. Pharmaceutical distribution remuneration in Europe. *J of Pharm Policy and Pract* **8** (Suppl 1), P23 (2015). https://doi.org/10.1186/2052-3211-8-S1-P23

Vogler S, Vitry A, Babar Z. Cancer drugs in 16 European countries, Australia, and New Zealand: A cross-country price comparison study. *The Lancet Oncology*,2016;17 1:47. https://doi.org/10.1016/S1470-2045(15)00449-0

Vogler S. How large are the differences between originator and generic prices? Analysis of five molecules in 16 European countries. *Farmeconomia. Health economics and therapeutic pathways* 2012; 13(Suppl 3): 29-41 2012; 13:41. Available from: https://www.semanticscholar.org/paper/4250caf4fd5fcd521db79316927d3f1d44a4a511

Wouters, O.J., Kanavos, P.G. (2017). A comparison of generic drug prices in seven European countries: a methodological analysis. *BMC Health Serv Res* **17**, 242 (2017). https://doi.org/10.1186/s12913-017-2184-5

Zaprutko T, Kopciuch D, Kus K, Merks P, Nowicka M, Augustyniak I, et al. Affordability of medicines in the European Union. 2017;12. https://doi.org/10.1371/journal.pone.0172753

Reason for exclusion: audit and feedback studies (n=15)

Colquhoun, H. L., Carroll, K., Eva, K. W., Grimshaw, J. M., Ivers, N., Michie, S., & Brehaut, J. C. (2021). Informing the research agenda for optimizing audit and feedback interventions: results of a prioritization exercise. *BMC medical research methodology*, *21*, 1-8. https://doi.org/10.1186/s12874-020-01195-5

Colquhoun, H., Michie, S., Sales, A., Ivers, N., Grimshaw, J. M., Carroll, K., ... & Brehaut, J. (2017). Reporting and design elements of audit and feedback interventions: a secondary review. *BMJ quality & safety*, *26*(1), 54-60. http://dx.doi.org/10.1136/bmjqs-2015-005004

Cooke, L. J., Duncan, D., Rivera, L., Dowling, S. K., Symonds, C., & Armson, H. (2018). How do physicians behave when they participate in audit and feedback activities in a group with their peers?. *Implementation Science*, *13*(1), 1-9. https://doi.org/10.1186/s13012-018-0796-8

Cooke, L. J., Duncan, D., Rivera, L., Dowling, S. K., Symonds, C., & Armson, H. (2018). The Calgary Audit and Feedback Framework: a practical, evidence-informed approach for the design and implementation of socially constructed learning interventions using audit and group feedback. *Implementation Science*, *13*(1), 1-18. https://doi.org/10.1186/s13012-018-0829-3

Glenngård, A. H., & Anell, A. (2021). The impact of audit and feedback to support change behaviour in healthcare organisations-a cross-sectional qualitative study of primary care centre managers. *BMC Health Services Research*, *21*(1), 1-12. https://doi.org/10.1186/s12913-021-06645-4

Grimshaw, J. M., Ivers, N., Linklater, S., Foy, R., Francis, J. J., Gude, W. T., & Hysong, S. J. (2019). Reinvigorating stagnant science: implementation laboratories and a meta-laboratory to efficiently advance the science of audit and feedback. *BMJ quality & safety*, *28*(5), 416-423. http://dx.doi.org/10.1136/bmjqs-2018-008355

Gude, W. T., van Engen-Verheul, M. M., van der Veer, S. N., de Keizer, N. F., & Peek, N. (2017). How does audit and feedback influence intentions of health professionals to improve practice? A laboratory experiment and field study in cardiac rehabilitation. *BMJ Quality & Safety*, *26*(4), 279-287. http://dx.doi.org/10.1136/bmjqs-2015-004795

Gude, W. T., Roos-Blom, M. J., van der Veer, S. N., Dongelmans, D. A., de Jonge, E., Francis, J. J., ... & de Keizer, N. F. (2018). Health professionals' perceptions about their clinical

performance and the influence of audit and feedback on their intentions to improve practice: a theory-based study in Dutch intensive care units. *Implementation Science*, *13*(1), 1-11. https://doi.org/10.1186/s13012-018-0727-8

Gude, W. T., van der Veer, S., van Engen-Verheul, M., de Keizer, N., & Peek, N. (2015, January). Inside the Black Box of Audit and Feedback: a Laboratory Study to Explore Determinants of Improvement Target Selection by Healthcare Professionals in Cardiac Rehabilitation.
In *MedInfo* (pp. 424-428). https://doi.org/10.3233/978-1-61499-564-7-424

Hartley, S., Foy, R., Walwyn, R. E., Cicero, R., Farrin, A. J., Francis, J. J., ... & AFFINITIE programme. (2017). The evaluation of enhanced feedback interventions to reduce unnecessary blood transfusions (AFFINITIE): protocol for two linked cluster randomized factorial controlled trials. *Implementation science*, *12*, 1-11. https://doi.org/10.1186/s13012-017-0614-8

Patel, S., Rajkomar, A., Harrison, J. D., Prasad, P. A., Valencia, V., Ranji, S. R., & Mourad, M. (2018). Next-generation audit and feedback for inpatient quality improvement using electronic health record data: a cluster randomised controlled trial. *BMJ Quality & Safety*, *27*(9), 691-699. http://dx.doi.org/10.1136/bmjqs-2017-007393

Scherer, F. M. (1993). Pricing, profits, and technological progress in the pharmaceutical industry. *Journal of Economic Perspectives*, *7*(3), 97-115. https://doi.org/10.1186/s43058-021-00155-4

Reszel, J., Dunn, S. I., Sprague, A. E., Graham, I. D., Grimshaw, J. M., Peterson, W. E., ... & Walker, M. C. (2019). Use of a maternal newborn audit and feedback system in Ontario: a collective case study. *BMJ Quality & Safety*, *28*(8), 635-644. http://dx.doi.org/10.1136/bmjqs-2018-008354

Roos-Blom, M. J., Gude, W. T., De Jonge, E., Spijkstra, J. J., Van Der Veer, S. N., Peek, N., ... & De Keizer, N. F. (2019). Impact of audit and feedback with action implementation toolbox on improving ICU pain management: cluster-randomised controlled trial. *BMJ quality & safety*, *28*(12), 1007-1015. http://dx.doi.org/10.1136/bmjqs-2019-009588

Reason for exclusion: technical analysis studies (n=10)

Comanor, W. S. (1965). Research and technical change in the pharmaceutical industry. *The Review of Economics and Statistics*, 182-190. https://doi.org/10.2307/1924065

Hartmann FGH, Maas VS. The effects of uncertainty on the roles of controllers and budgets: An exploratory study. 2011;41(5):458. *Accounting and Business Research*, 41:5, 439-458. https://doi.org/10.1080/00014788.2011.597656

Herweg F, Müller D. A comparison of regret theory and salience theory for decisions under risk. 2021;193,1,105226. https://doi.org/10.1016/j.jet.2021.105226.

Ivers, N. M., & Barrett, J. (2018). Using report cards and dashboards to drive quality improvement: lessons learnt and lessons still to learn. *BMJ Quality & Safety*, *27*(6), 417-420. http://dx.doi.org/10.1136/bmjqs-2017-007563

Liozu SM, Hinterhuber A. Pricing orientation, pricing capabilities, and firm performance. *Management Decision*, Vol. 51 No. 3, pp. 594-614. https://doi.org/10.1108/00251741311309670

Malmi T, Granlund M. (2009). In search management accounting theory. *European Accounting Review,*18(3), 597-620. https://doi.org/10.1080/09638180902863779

Mhlanga BS, Suleman F. Price, availability and affordability of medicines. 2014;6. *African Journal of Primary Health Care & Family Medicine* , *6*(1), 1-6. Retrieved November 20, 2023, from http://www.scielo.org.za/scielo.php?script=sci_arttext&pid=S2071-29362014000100014&lng=en&tlng=.

Popesko B, Tučková Z. Utilization of process-oriented costing systems in healthcare organizations. (2012). *INTERNATIONAL JOURNAL OF MATHEMATICAL MODELS AND METHODS IN APPLIED SCIENCES*. 1(6). https://www.semanticscholar.org/paper/ad7736b9be1ad0963d4f5561cf9b9a9fbf6875ed

Smith, G.E. and Woodside, A.G. (2009), "Chapter 9 Pricing theory and practice in managing business-to-business brands", Glynn, M.S. and Woodside, A.G. (Ed.) *Business-To-Business Brand Management: Theory, Research and Executivecase Study Exercises* (*Advances in Business Marketing and Purchasing, Vol. 15*), Emerald Group Publishing Limited, Bingley, pp. 429-486. https://doi.org/10.1108/S1069-0964(2009)0000015013

Vernon J, Gusen P. Technical Change and Firm Size: The Pharmaceutical Industry. 1974; 56:302. *The Review of Economics and Statistics*, Vol. 56, No. 3 (Aug., 1974), pp. 294-302 (9 pages). https://doi.org/10.2307/1923966

Reason for exclusion: quality improvement studies n=2

Conn LG, Hoeft CJ, Neal ML, Nathens A. Use of performance reports among trauma medical directors and programme managers in the American College of Surgeons' Trauma Quality Improvement Program: a qualitative analysis. 2019; 28(9):728. *BMJ Quality & Safety* http://dx.doi.org/10.1136/bmjqs-2018-008797

Scarpis E, Brunelli L, Tricarico P, Poletto M, Panzera A, Londero C, et al. (2021). How to assure the quality of clinical records? A 7-year experience in a large academic hospital. 16. *PLOS ONE*. https://doi.org/10.1371/journal.pone.0261018

Reason for exclusion: price referencing studies (n=33)

Brekke KR, Holmås T, Straume O. Reference Pricing, Competition, and Pharmaceuti Scarpis E, Brunelli L, Tricarico P, Poletto M, Panzera A, Londero C, et al. (2021). How to assure the quality of clinical records? A 7-year experience in a large academic hospital. *PLOS ONE* 16. https://doi.org/10.1371/journal.pone.0261018

Brekke, K. R., Holmas, T. H., & Straume, O. R. (2011). Reference pricing, competition, and pharmaceutical expenditures: theory and evidence from a natural experiment. *Journal of Public Economics*, *95*(7-8), 624-638. https://doi.org/10.1016/j.jpubeco.2010.11.015.

Csanádi M, Kaló Z, Prins CPJ, Grélinger E, Kiss AM, Fricke F, et al. The implications of external price referencing on pharmaceutical list prices in Europe. 2018. *Health Policy and Technology*,7(3),pp. 243-250. https://doi.org/10.1016/j.hlpt.2018.07.005.

Danzon P, Ketcham JD. (2003). Reference Pricing of Pharmaceuticals for Medicare: Evidence From Germany, the Netherlands, and New Zealand. *Forum for Health Economics & Policy, 7*(1). https://doi.org/10.2202/1558-9544.1050

Dylst, P., Vulto, A., & Simoens, S. (2012). Reference pricing systems in Europe: characteristics and consequences. *Generics and Biosimilars Initiative Journal*, *1*(3-4), 127-131. https://doi.org//10.5639/gabij.2012.0103-4.028

Fontrier, AM., Gill, J. & Kanavos, P. International impact of external reference pricing: should national policy-makers care?. (2019). *Eur J Health Econ* 20, 1147–1164. https://doi.org/10.1007/s10198-019-01083-w

Galizzi M, Ghislandi S, Miraldo M. (2011). Effects of Reference Pricing in Pharmaceutical Markets.29:33. *Pharmacoeconomics* **29**, 17–33. https://doi.org/10.2165/11537860-000000000-00000

Ghislandi S, Armeni P, Jommi C. (2013)The impact of generic reference pricing in Italy, a decade On, 14:969. *Eur J Health Econ* **14**, 959–969. https://doi.org/10.1007/s10198-012-0442-3

Gill J, Fontrier A-M, Kyriopoulos D, Kanavos P. (2019). Variations in external reference pricing implementation: does it matter for public policy? 2019; 20:1397. *Eur J Health Econ* **20**, 1375–1397. https://doi.org/10.1007/s10198-019-01100-y

Grootendorst P, Stewart D. A re-examination of the impact of reference pricing on anti-hypertensive drug plan expenditures in British Columbia. 2006;15 7:742. **Health Econ.15: 735–742 (2006).** https://doi.org/10.1002/hec.1103

Schneider, P., Habl, C., Németh, G., Primožič, S., & Šebesta, R. (2017). EURIPID Best practice report on External Reference Pricing (ERP). *Brussels: Health Programme of the European*

Union.23. Available from: https://www.semanticscholar.org/paper/632a04d857f38e57aff4bb27e0b2986fe11dced9
Vogler, S. (2019). Assessment of External Price Referencing and Alternative Policies. In *Medicine Price Surveys, Analyses and Comparisons* (pp. 369-419). Academic Press. https://doi.org/10.1016/B978-0-12-813166-4.00019-X

Reason for exclusion: cost-containment studies (n=4)

Carone, G., Schwierz, C., & Xavier, A. (2012). Cost-containment policies in public pharmaceutical spending in the EU. Available at SSRN: https://ssrn.com/abstract=2161803 or http://dx.doi.org/10.2139/ssrn.2161803
Kemp R. Australia's "free ride" in pharmaceuticals: can it last? 1996;19 1:94. Kemp, R. (1996). Australia's" free-ride" in pharmaceuticals: Can it last?. *Australian Health Review*, *19*(1), 81-94. https://doi.org/10.1071/AH960081
Morgan, S. G., Vogler, S., & Wagner, A. K. (2017). Payers' experiences with confidential pharmaceutical price discounts: a survey of public and statutory health systems in North America, Europe, and Australasia. *Health Policy*, *121*(4), 354-362. https://doi.org/10.1016/j.healthpol.2017.02.002
Verbeeten FH m. public sector cost management practices in The Netherlands (2011). *International Journal of Public Sector Management*, 24(6), pp. 492-506. https://doi.org/10.1108/09513551111163620

Reason for exclusion: research and development cost studies (n=4)

Grabowski, H., & Vernon, J. (1990). A new look at the returns and risks to pharmaceutical R&D. *Management Science*, *36*(7), 804-821. https://doi.org/10.1287/mnsc.36.7.804
Leffler, K. B. (1981). Persuasion or information? The economics of prescription drug advertising. *The Journal of Law and Economics*, *24*(1), 45-74.
Wosinska, M. E. (2002). *The economics of prescription drug advertising* (Order No. 3063603). Available from ProQuest Central. (251731633). Retrieved from https://www.proquest.com/dissertations-theses/economics-prescription-drug-advertising/docview/251731633/se-2
Schnee J.E.(1979). R&{D} strategy in the U.S. pharmaceutical industry., *Research Policy*,8(4),1979,pp.364-382. https://doi.org/10.1016/0048-7333(79)90003-9.
Schwartzman D. (1978). "Innovation in the Pharmaceutical Industry". Br Med J. 8;1(6117):919. https://doi.org/10.1136/bmj.1.6117.919-a.

***Appendix* 4*:* Summary of sources that discussed equivalent units of production concept for teaching or survey without explicit unit cost content.**

Author(s)	Title	Brief description	Method	Findings
Etnier, D. (1961)	Teaching equivalent Production with a Chart	Attempt to memorize formula and failure to grasp basic concept of what equivalent production is identified as reason for students'challenges with the concept	Use of chart to demonstate	2 formulae same result
Simionescu, S. (2013)	Reviews on Accounting of Revenues Associated to the Production Cost	Paper discusses how to separate costs of the finished products already marked up for production in progress, using the stock assessment methods, in IAS 2, respectively the weighted	IAS 2 Stock assessment methods	

	of the Work in Progress	average cost method (WAC), the FIFO method and the standard cost method, methods used in theory and practice exclusively for evaluating stocks.		
DeCoster, T. (1964)	The Unit Cost Denominator in Process Costing	Suggests how to solve the problem of determining the unit cost of the goods remaining in the final inventory.	Periodic inventory concept of determining unit cost of goods remaining in closing inventory.	Students' challenges are lessened when all impacts are viewed through the costing of the final inventory using the periodic inventory concept.
Silvester et al. (2013)	Cost Flow Diagrams as an Alternative Method of External Problem Representation - A Diagrammatic Approach to Teaching Cost Accounting and Evidence of Its Effectiveness	Provides statistical evidence on the ease of using an alternative new visually based diagrammatic approach based on cost flow diagrams to represent external problem.	Diagrammatic solutions to three different traditional cost accounting problems	When a diagrammatic style is used, student performance improves significantly.
Zhao and Huang (2016)	Cost Accounting Teaching: Focus on the Processes and Operations	All pricing methods, such as the cost plus method, target profits method, psychological pricing method, and so on, are based on exact cost information.<br><br>To describe and separate basic production costs into unfinished products and finished products, the core ideas of process management are introduced.	Use of process management to explain cost accounting	The teaching modality focused on processes and operations outperforms the traditional method.
Buchheit et al. (2002)	A Note on Equivalent Units Calculations	An investigation into the relative effectiveness of two different methods for teaching process costing equivalent units problems.	A quasi-experiment conducted on 431 undergraduate cost accounting students	Students perform better with time-based approach than the traditional rule-based approach in literature
Kelly and Shoemaker. (2018)	Closing Pandora's Box: Reducing Students'	Simulation approach used in classroom setting to explain to undergraduates in both	Experimental design	Approach applies to weighted-

	Confusion With A Process Costing Simulation	introductory managerial accounting and cost accounting courses the principles of process costing and how to apply these principles to a problem.		average or first-in, first-out (FIFO) methods of process costing respectively.

Source: Authors
